# Generating realistic scaled complex networks

**DOI:** 10.1007/s41109-017-0054-z

**Published:** 2017-10-13

**Authors:** Christian L. Staudt, Michael Hamann, Alexander Gutfraind, Ilya Safro, Henning Meyerhenke

**Affiliations:** 1 Laboratory for Mathematical Analysis of Complexity and Conflicts, University of Illinois at Chicago, Chicago, IL USA; 2 Institute of Theoretical Informatics, Karlsruhe Institute of Technology (KIT), Karlsruhe, Germany; 3 School of Computing, Clemson University, Clemson, SC USA

**Keywords:** Network generation, Multiscale modeling, Network modeling, Communities

## Abstract

Research on generative models plays a central role in the emerging field of network science, studying how statistical patterns found in real networks could be generated by formal rules. Output from these generative models is then the basis for designing and evaluating computational methods on networks including verification and simulation studies. During the last two decades, a variety of models has been proposed with an ultimate goal of achieving comprehensive realism for the generated networks. In this study, we (a) introduce a new generator, termed ReCoN; (b) explore how ReCoN and some existing models can be fitted to an original network to produce a structurally similar replica, (c) use ReCoN to produce networks much larger than the original exemplar, and finally (d) discuss open problems and promising research directions. In a comparative experimental study, we find that ReCoN is often superior to many other state-of-the-art network generation methods. We argue that ReCoN is a scalable and effective tool for modeling a given network while preserving important properties at both micro- and macroscopic scales, and for scaling the exemplar data by orders of magnitude in size.

## Introduction

Networks are widely used to represent connections between entities, because they provide intuitive windows into the function, dynamics, and evolution of natural and man-made systems. However, high-quality, large-scale network data is often unavailable because of economic, legal, technological, or other obstacles (Chakrabarti and Faloutsos 2006; Brase and Brown 2009). For example, human contact networks in the context of infectious disease spread are notoriously difficult to estimate, and thus our understanding of the dynamics and control of epidemics stems from models that make highly simplifying assumptions or simulate contact networks from incomplete or proxy data (Eubank et al. 2004; Keeling and Rohani 2008; Meyers et al. 2005). In another domain, the development of cybersecurity systems requires testing across diverse threat scenarios and validation across diverse network structures that are not yet known, in anticipation of the computer networks of the future (Dunlavy et al. 2009). In both examples, the systems of interest cannot be represented by a single exemplar network, but must instead be modeled as collections of networks in which the variation among them may be just as important as their common features. Such cases point to the importance of data-driven methods for synthesizing networks that capture both the essential features of a system and realistic variability in order to use them in such tasks as simulations, analysis, and decision making.

A good network generator must meet two primary criteria: realism and diversity. The first, realism, needs to consider any properties of the network that govern the domain-specific processes of interest such as system function, dynamics, and evolution. Hence, realism may depend on both structural network features and the more subtle emerging features of the network. Consider the following examples in potential applications:

- Models of social networks should be able not only to reproduce structural features such as small-world properties, but also, and perhaps more importantly, to emulate emergent sociological phenomena such as interactions between individuals in a community, as driven by their psychological needs and daily routines. That is, the generated network should show similar interactions by its artificial individuals, as determined by implicit psychological and social rules.
- Models of connected solar energy collectors of different sizes and capacities should simulate realistic energy outputs influenced by the weather.
- Models of metabolic interactions should ultimately reflect biochemical properties of a cell.

Second, a synthetic network should reflect naturally occurring stochasticity in a system, without systematic bias that departs from reality. This feature is important for benchmarking and evaluating the robustness of network-based algorithms, anonymizing networks, and generating plausible hypothetical scenarios. In particular, when engineering algorithms, the ability to create good synthetic test data sets is valuable to estimate effectiveness and scalability of the proposed methods.

In addition, a network generator should be effective in tasks such as obfuscation (replacing restricted real data with similar synthetic data), compression (storing only a generator and its parameters instead of large graphs), as well as extrapolation and sampling (generating data at larger or smaller scales). Finally, the running time and memory requirements of the generator should be acceptable for realistically large datasets - datasets that may include millions or even billions of nodes and edges.

### Problem definition

We envision the following usage scenarios. Given is an original (or real) network *O*=(*V*,*E*) (*n*_*o*_:=|*V*|, and *m*_*o*_:=|*E*|) that, for example, cannot be freely shared. We would like to be able to create a synthetic network *R* (with *n*_*r*_ nodes) that matches the original in essential structural properties, so that computational results obtained from processing this network are representative for what the original network would yield. We refer to *R* as a *replica*. We assume that whoever creates the replica has access to *O* and can pass it to a *model fitting* algorithm which uses it to parametrize a generative model.

More importantly, in addition to producing *scale-1 replicas* (where *n*_*r*_=*n*_*o*_), in the second scenario we want to use the generative model for *extrapolation*, i.e., we would like to parametrize it to produce a *scaled replica* *R* ^*x*^ that has *n*_*r*_=*x*·*n*_*o*_ nodes, where *x* is called the *scaling factor*. The structural properties of *R* ^*x*^ should be such that they resemble a later growth stage of the original (also see Section “Realistic replicas”). This should enable users of the replica to extrapolate the behavior of their methods when the network data is significantly scaled.

Finally, with respect to performance, we would like the generator algorithm and implementation as well as the fitting scheme to be efficient enough to produce large data sets (on the order of several millions of nodes and edges) quickly in practice.

### Related work

A number of network generation methods have been developed, and these fall into two classes: generative models and editing methods (see surveys in Goldenberg et al. (2010); Chakrabarti and Faloutsos (2006); Brase and Brown (2009); Dunlavy et al. (2009)). The first set of methods produces networks (by using such elementary operations as randomization and replication) from small initial seed networks (sometimes empty). The goal of such generation is to produce the structure that matches real data in *prespecified* properties, such as the degree distribution (Albert and Barabási 2002; Newman 2003; Mahadevan et al. 2006), clustering (Bansal et al. 2009), and the number of small subgraphs. These methods are attractive because they often produce networks with the desired features and are grounded in well-developed theory (e.g., Erdos and Renyi (1960)). Some of these graph generators mechanistically model network growth (Barabási and Albert 1999; Krapivsky and Redner 2001; Leskovec et al. 2008), whereas others incorporate domain-specific information such as geographic location (Watts and Strogatz 1998) and cyber networks topological properties (Medina et al. 2000; Dunlavy et al. 2009).

One of the most successful generative strategies is based on Kronecker graphs (Leskovec et al. 2010) (including stochastic Kronecker graphs; see also related work (Chakrabarti and Faloutsos 2006; Palla et al. 2010)). Graphs generated by this model preserve properties such as degree distribution, diameter, and some eigenvalues. Generative methods often describe an evolutionary process that can potentially lead to the original network; however, the probability that it will lead to the structure that is structurally similar (or approximately isomorphic) to the original one at some coarse-grained resolutions (but not necessarily at finest) is usually negligible. This makes generative methods ill-suited for studies such as simulations when one may need to work with systems that are similar to the original. For example, in the context of supply chain network simulations (Terzi and Cavalieri 2004) (and other engineered or man-made systems), a realism of a network can be described (though, typically, not formally) in terms of the supply chain functionality which is in turn related to the structure of domain specific coarse-grained resolutions. Several generative models admit fast generators and are thus in our focus. Among those models are R-MAT (Chakrabarti et al. 2004), BTER (Kolda et al. 2013), and Hyperbolic Unit Disk Graphs (Krioukov et al. 2010). However, fitting the models’ parameters so as to replicate a wide range of properties is far from being satisfactory so far. For example, a previous fitting scheme by Leskovec et al. (2007) for R-MAT graphs is quite time-consuming already for medium-sized networks (Staudt 2016).

The other class of network generators, graph editing (Mihail and Zegura 2003), starts with a given (real or empirical) network and randomly changes its components until the network becomes sufficiently different than the original network. These are designed to introduce variability while preserving key structural properties. The multiscale network generator MUSKETEER (Gutfraind et al. 2015) is an editing approach that is able to produce highly realistic synthetic networks by applying a local editing at different coarse-grained resolutions. The approach is comparable to ReCoN in the quality of generated networks; however, its implementation (Gutfraind et al. 2012) requires further work to be applicable on very large networks.

### Outline and contribution

In this paper we develop and evaluate a fast generator that focuses on creating realistic *scaled* replicas of complex networks. We point out in “Realistic replicas” section which criteria we consider important for calling a (scaled) replica realistic. In particular we conceptualize realism in two ways: (i) matching an original graph in a set of important structural properties, and (ii) matching the running time behavior of various graph algorithms. Typically, being an important applied criteria, the last is not considered by most existing network generators.

Our new generator ReCoN (short for *Replication of Complex Networks*) is described in “The generation algorithm ReCoN” section. It uses and extends ideas of LFR, a generator used for benchmarking community detection algorithms. Using the original degrees and a discovered community structure, we are able to capture a much more detailed signature of the network than a parametrization of the LFR generator. In “Fitting generative models to input graphs” section and “Considered models” section we discuss the generative models that we use for comparison (R-MAT, Hyperbolic Unit Disk Graph, and BTER are among them) and develop model fitting schemes for them.

Our comparative experimental study in “Computational experiments” section indicates that ReCoN performs overall quite well and usually better than other generators in terms of realism. We can also conclude that the ReCoN implementation is fast, as it is capable of creating realistic scaled replicas on the scale of 10^8^ edges in minutes. The ReCoN code is publicly available in the open-source network analysis package NetworKit (Staudt et al. 2016). This paper is based on a preliminary conference version (Staudt et al. 2016). It was extended in particular by additional experimental results (including scaling experiments), revised fitting schemes, and by a detailed discussion of open problems and promising research directions (“Multi-scale modeling and open problems” section).

## Realistic replicas

We consider a generative model realistic if there is high structural similarity between the synthetic graphs produced and relevant real-world networks. It is neither our goal nor generally desirable to obtain an exact correspondence between original and replica. First, this would exclude the use case of obfuscation. Secondly, obtaining an isomorphic graph is rarely required for typical experiments. Note that we consider a single “realism score” for each model inappropriately reductionist. Instead, we quantify diverse aspects of realism in our experimental evaluation and leave it to the reader to decide about their relative importance.

For scale-1 replicas (with the same size as the original), we measure the similarity in terms of a set of commonly used metrics: Sparsity (number of edges vs number of nodes); degree distribution (more precisely its Gini coefficient); maximum degree as a proxy for the connectedness of hub nodes; average local clustering coefficient to measure the local presence of triangles; diameter to monitor the small-world effect; number of connected components and number of communities as additional non-local features. These metrics cover both local and global properties and are deemed important characteristics of networks (Newman 2010).

**Fig. 1:**
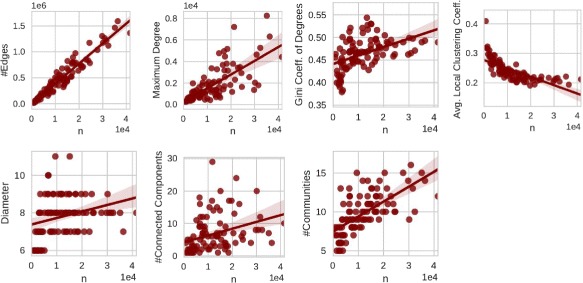
Scaling behavior of 100 Facebook networks; from left to right and top to bottom: number of edges, maximum degree, Gini coefficient of degree distribution, average local clustering coefficient, diameter, number of components, number of communities found by Parallel Louvain Method

How can we extend the notion above regarding realism to *scaled* replicas of a network? To answer this question, let us look at the scaling behavior of a set of 100 Facebook social networks (Traud et al. 2012). These networks were collected in September 2005 at an early stage of the Facebook online social networking service in which networks were still separated by universities. The nodes of each network are members of a (single) US university or college. Most of them are students, and the edges represent friendships on Facebook. Since these networks were formed by the local actions of a collection of social actors which have essentially similar behavior, the mechanism of growth by which the networks have assembled themselves is essentially the same. These networks of different sizes can in a sense be treated as differently scaled versions of “the” Facebook social network. In Fig. 1 we demonstrate basic structural measures of these Facebook networks against the number of nodes *n*, as well as a regression line and confidence intervals (shaded area) to emphasize the trend. While linear regression may not always seem completely appropriate for these data, the general trend is typically still captured.

We can observe from Fig. 1 a growth of the number of edges *m* that is linear in *n*, an increase in the skew of the node degree distribution as measured by the Gini coefficient, a growing maximum node degree, a slightly falling average local clustering coefficient, a nearly constant small diameter of the largest connected component, and a slightly growing number of connected components (which can be explained by some small connected components that exist in addition to a giant component). We detect communities using PLM (Parallel Louvain Method), a modularity-driven community detection heuristic (Staudt and Meyerhenke 2016), and report the number of communities minus the number of these small connected components. It can be observed that the number of non-trivial communities grows slightly.

While we do not claim that these scaling laws are universal, the trends represented here are commonly observed (Caldarelli and Vespignani 2007; Boccaletti et al. 2006; Snijders 2001). Thus, we use them to define desired scaling properties for the remainder of the study as follows: *m* grows linearly with *n*; the diameter does not change significantly, preserving the “small world property”; the shape of the degree distribution remains skewed; the maximum node degree increases; the number of connected components may grow; the number of communities increases slightly.

Recall that one use case for our generator is testing graph and network analysis algorithms. Since the running time is an essential feature in such tests, we also consider a realistic replication of running times important. To this end, we select a set of graph algorithms that (i) compute important features of networks and are thus frequently used in network analysis tasks and that (ii) cover a variety of patterns of computation and data access, each of which may interact differently with the graph structure. The set consists of algorithms for connected components (essentially breadth-first search), PageRank (via power iteration), betweenness approximation (according to Geisberger et al. (2008)), community detection (Parallel Louvain Method, Staudt and Meyerhenke (2016)), parallel core decomposition (according to Dasari et al. (2014)), parallel triangle counting (according to Hamann et al. (2016)), and spanning forest (Kruskal’s algorithm with random edge weights).

## The generation algorithm ReCoN

We introduce ReCoN, a generator for replicating and scaling complex networks. Its input is a graph and a community structure on it. For fitting a given graph without given community structure, we use Parallel Louvain Method (PLM) (Staudt and Meyerhenke 2016) in order to detect a community structure first. It is a very fast and effective greedy algorithm for modularity maximization. *The basic idea of* ReCoN *is to randomize the edges inside communities and the edges between communities while keeping the node degrees.* This happens separately such that each community keeps exactly as many edges as it had before. For scaling a graph, we first create as many disjoint copies of the graph as desired and then apply the aforementioned steps. During the randomization of the edges between the communities, the copies usually become connected with each other.

The idea of randomizing graphs inside and between communities is inspired by the LFR generator, a benchmark graph generator for community detection algorithms (Lancichinetti et al. 2008) in which the basic building blocks are also a random subgraph per community and a global graph. However, in the LFR generator, the degrees and communities are not given but generated using a power law degree distribution and a power law community size distribution with nodes assigned to communities at random, while ReCoN uses the given graph as input for them.

For randomizing graphs while preserving the degree sequence, we use random edge switches where two edges {*u*,*v*}, {*y*,*z*} chosen uniformly at random are changed into {*u*,*z*}, {*y*,*v*} if the resulting graph is still simple, i.e. does not contain any duplicate edges or self-loops. Similarly to the edge switching implementation provided by (Viger and Latapy 2005) we use 10 times the number of edges as the number of random edge switches. Previously performed experiments (e. g., Milo et al. 2003) have shown that this is enough to expect the resulting graph to be drawn uniformly at random from all graphs with the given degree sequence.

For an original graph *O*=(*V*,*E*) with *n*_*o*_=|*V*| nodes and a desired scaling factor *x*, ReCoN executes the following steps:

1. Detect a community structure $\mathcal {C} = \{C_{1}, \dots, C_{k}\}$ on *O* using Parallel Louvain Method.
2. Generate graph *H* as the disjoint union of *x* copies of *O*. The community structure is also copied such that the new community structure $\mathcal {D} = \{D_{1}, \dots, D_{x \cdot k}\}$ consists of *x*·*k* communities, i.e., each copy of *O* gets its own copy of the community structure that is aligned with the structure of the copied graph.
3. For each community *D*_*i*_, 1≤*i*≤*x*·*k*, randomize the edges of the subgraph *H*[*D*_*i*_] that is induced by the community *D*_*i*_ while keeping the degree distribution using random edge switches.
4. Randomize the remaining edges, i. e., all edges in *H* that are not part of one of the subgraphs *H*[*D*_*i*_], using random edge switches. Note that afterwards some edges that were not in one of the *H*[*D*_*i*_] can now be inside a community. In order to avoid this, rewiring steps are performed by executing edge switches of such forbidden edges with random partners. A similar step is also used in the LFR generator, where it was observed that, in practice, only few rewiring steps are necessary (Lancichinetti and Fortunato 2009).

Note that it is not necessary to start with the full information of the original graph in Steps 3 and 4. It is actually sufficient to know a community structure (as opposed to the whole original graph) and for each node the internal and external degree, i.e, how many neighbors it has inside and outside its community, respectively. For our implementation we choose this alternative. Further, we execute Step 3 in parallel for all communities as the subgraphs are disjoint.

Depending on the domain in which the network generation is required, the Parallel Louvain Method can be replaced with another method and even objective. It is well known that modularity objective is not necessarily the perfect maximization objective in some areas, so this method can potentially be replaced with something else in Step 1 that would not violate the performance requirements of the generator.

In addition to replicating important properties with high fidelity, the randomization in Steps 3 and 4 naturally produces random variance among the set of replicas.

### Fitting generative models to input graphs

Parametrized generative models require learning model input parameters from the original network. A fitting scheme is an algorithm that takes a network as input and estimates parameters of a generative model. Because, usually, such schemes are not unique, exploring them would be important future work. For this study, we have chosen one straightforward scheme per model, parameters of which are summarized in Table 1. Below we discuss a fitting scheme for power law degree distributions, and briefly describe the generative models that are compared with ReCoN.

**Table 1:** Parameters set to fit a model to a given graph, and to produce a scaled-up replica

Model	Parameters	Fitting	Fitting scaling by $x \in \mathbb {N}$
Erdős–Rényi	*E* *R*(*n* ^′^,*p*)	*n* ^′^=*n* $p = \frac {2m}{n \cdot (n-1)}$	*n* ^′^=*x*·*n* $p = \frac {2m}{x \cdot n \cdot (n-1)}$
Barabasi-Albert	*B* *A*(*n* ^′^,*k*)	*n* ^′^=*n* *k*=⌊*m*/*n*⌋	*n* ^′^=*x*·*n* *k*=⌊*m*/*n*⌋
Chung-Lu	*C* *L*(*d*)	*d*=(deg(*u*))_*u*∈*V*_	$d = \cup _{i=1}^{x} (\deg (u))_{u \in V}$
Edge-Switching Markov Chain	*E* *M* *C*(*d*)	*d*=(deg(*u*))_*u*∈*V*_	$d = \cup _{i=1}^{x} (\deg (u))_{u \in V}$
R-MAT	*R* *M*(*s*,*e*,(*a*,*b*,*c*,*d*))	*s*=⌈log2*n*⌉ *e*=⌊*m*/*n*⌋ (*a*,*b*,*c*,*d*)=kronfit(*O*)	*s*=⌈log2*x*·*n*⌉ *e*=⌊*m*/*n*⌋ (*a*,*b*,*c*,*d*)=kronfit(*O*)
Hyperbolic Unit-Disk	$HUD(n, \bar {d}, \gamma)$	*n*=*n* $\bar {d} = 2 \cdot (m / n)$ *γ*= max{2.1,plfit((deg(*u*))_*u*∈*V*_)}	*n*=*x*·*n* $\bar {d} = 2 \cdot (m / n)$ *γ*= max{2.1,plfit((deg(*u*))_*u*∈*V*_)}
BTER	*B* *T* *E* *R*(*d*,*c*)	$d = (n_{d})_{d \in (0, \dots, d_{\max })}$ $c = (c_{d})_{d \in (0, \dots, d_{\max })} $	$d = (n_{d} \cdot x)_{d \in (0, \dots, d_{\max })}$ $c = (c_{d})_{d \in (0, \dots, d_{\max })} $
LFR	$LFR(n',{\newline } \gamma,\bar {d},d_{\max }, {\newline } \beta, c_{\min },c_{\max })$	*n* ^′^=*n* *γ*,*d*_min_,*d*_max_=plfit((deg(*u*))_*u*∈*V*_) *ζ*_*s*_={|*C*| | *C*∈PLM(*O*)} *β*,*c*_min_,*c*_max_=plfit*(*ζ*_*s*_)	*n* ^′^=*x*·*n* *γ*,*d*_min_,*d*_max_=plfit((deg(*u*))_*u*∈*V*_) *ζ*_*s*_={|*C*| | *C*∈PLM(*O*)} *β*,*c*_min_,*c*_max_=plfit*(*ζ*_*s*_)

We consider an original graph *O*=(*V*,*E*) with *n*_*o*_=|*V*|, *m*_*o*_=|*E*| and maximum degree *d*_max_. We denote as (*a*_*i*_)_*i*∈*M*_ a sequence of elements *a* with indices *i* from an (ordered) set *M*, so

$${\bigcup}_{j = 0}^{k} {\left({a}^{j}_{i}\right)}_{i \in M} $$

denotes the ordered concatenation of *k* sequences. The clustering coefficient of node *u* is denoted by c_*u*_. The number of nodes with degree *d* is denoted by

$$n_{d} := | V_{d} | = |\{v \in V: \deg(v) = d \} |, $$

and

$$c_{d} := \frac{1}{n_{d}} \sum_{u \in V_{d}} \textsf{c}_{u} $$

corresponds to the average clustering coefficient for nodes of degree *d*.

After a brief description of the considered models, we discuss fitting of power-law distributions, and continue with a discussion of the parametrization of each model.

### Considered models

Apart from ReCoN, we include the following models in our experimental study: *Erdős–Rényi* random graphs (Erdos and Renyi 1960), *Barabasi–Albert* (Albert and Barabási 2002), *Chung-Lu* (Aiello et al. 2000), *Edge-Switching Markov Chain*, *Recursive Matrix* (R-MAT) (Chakrabarti et al. 2004), *Hyperbolic Unit-Disk Graph* (Krioukov et al. 2010), *BTER* (Seshadhri et al. 2011), and *LFR* (Lancichinetti et al. 2008).

#### Fitting power law degree distribution

Both LFR and Hyperbolic Unit Disk Graph generators produce graphs with a power law degree distribution. Therefore, at least the power law exponent, and, in the case of the LFR generator, also the minimum and maximum degrees need to be determined such that the degree distribution fits the real network. In Table 1, plfit refers to our custom power law fitting scheme.

A practical replication of a network requires preserving the original average (otherwise, the density will be changed) as well as minimum and maximum degrees (applications can be sensitive to such fundamental properties as degree-1 nodes and the distribution of hubs).

In general, it is assumed (and implemented in many algorithms (Clauset et al. 2009)) that a power law degree distribution only holds from a certain degree on and that for smaller degrees, the distribution might be different. As the LFR generator only generates a plain power law degree distribution, we cannot apply this assumption. Therefore, we fit the power law degree distribution exponent such that, with the given minimum and maximum degree, the average degree of the real network is expected when a degree sequence is sampled from this distribution. Using binary search in the range of [−6,−1], we repeatedly calculate the expected average degree until the power law exponent is accurate up to an error of 10^−3^.

#### Erdős–Rényi, Barabasi-Albert, Chung-Lu and Edge-Switching Markov Chain

*Erdős–Rényi* random graphs (Erdos and Renyi 1960), while not considered similar to most real-world graphs, are fundamental and an important baseline. The Erdős–Rényi model does not provide many options for parametrization. The edge probability *p* is set to produce the same edge-to-node ratio $\frac {m}{n}$ as the original. The *Barabasi–Albert* model (Albert and Barabási 2002) implements a preferential attachment process by which a power law degree distribution emerges, which has been claimed to be a typical feature of real complex networks. In Barabasi–Albert model, we set the number of edges coming with each new node to fit the original edge-to-node ratio. The Chung-Lu model (Aiello et al. 2000) recreates a given degree sequence in expectation. The Edge-Switching Markov Chain model generates a graph that is randomly drawn from all graphs with exactly the given degree sequence (see e.g. (Milo et al. 2003; Schlauch et al. 2015)). In both Chung-Lu and Edge-Switching Markov Chain we use the original degree sequence. To generate larger networks, *x* copies of this sequence are concatenated, multiplying the number of nodes by *x* while keeping the relative frequency of each degree.

#### R-MAT

The R-MAT model (Chakrabarti et al. 2004) was proposed to recreate various properties of complex networks, including an optional power-law degree distribution, the small-world property and self-similarity. The R-MAT model can only generate graphs with 2^*s*^ nodes, where *s* is an integer scaling parameter. In order to target a fixed number of nodes *n*_*r*_, we calculate *s* so that 2^*s*^>*n*_*r*_ and delete 2^*s*^−*n*_*r*_ random nodes. We sample edges (ignoring self-loops and duplicates) until the average degree of the graph without the deleted nodes reaches the average degree of the original graph. The choice of the parameters *a*,*b*,*c*,*d* requires some discussion.

Leskovec et al. (2007) propose a method to “given a large, real graph […], generate a synthetic graph that matches its properties”, using stochastic Kronecker graphs: Starting with a 2-by-2 stochastic initiator matrix *I*, Kronecker products are calculated so that *I* ^*s*^ is a stochastic matrix of dimension 2^*s*^ that yields edge probabilities of a graph. This is equivalent to the R-MAT model as it yields the same edge probabilities. They attempt to fit model parameters so that the likelihood that a given original graph *O* was generated starting from an initiator matrix *I* is maximized, and propose the kronfit gradient descent algorithm that iteratively estimates *I* in *O*(*m*) time. They do not explicitly mention the case of creating a scaled replica, but it is clear that the method is capable of producing graphs for arbitrary exponents *s*. We use an implementation of kronfit which is distributed with the SNAP network analysis tool suite (Leskovec and Sosič 2014).

**Fig. 2:**
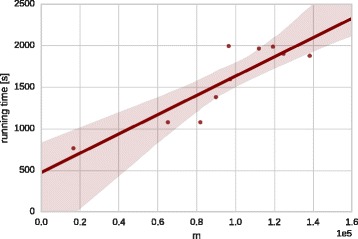
Running time of 50 iterations of the kronfit algorithm in relation to the number of edges *m* of the input network

The high running times of kronfit (see Fig. 2) mean that running kronfit on every network to be replicated is not practical if the collection of networks is large. For example, in our experiments with 100 Facebook networks, we estimate the R-MAT parameters as follows: We assume that the 100 Facebook networks constitute a class with essential structural commonalities, and run the recommended 50 iterations of kronfit on one typical network, fb-Caltech36. The resulting initiator is applied to replicate all Facebook networks. For other sets of networks, the assumption of structural similarity cannot be made, so we run the recommended 50 iterations of kronfit for each of them.

#### Hyperbolic unit disk graphs

The random hyperbolic graph model embeds nodes into hyperbolic geometry and connects close nodes with higher probability (Krioukov et al. 2010). The unit-disk variant of Hyperbolic Unit Disk Graphs we use in this paper connects only nodes whose distance is below a certain threshold. We are focusing on the unit-disk variant to be able to use a very fast generator for this model (von Looz et al. 2015). The model has been shown to replicate some properties observed in real networks, such as a power-law degree distribution. This method receives as parameters the desired number of nodes, the average degree of the original network and a power law exponent which is fitted as described above. As the given power law exponent must be larger than 2, we supply at least an exponent of 2.1.

#### BTER

The BTER model (Seshadhri et al. 2011) receives a degree distribution and the desired clustering coefficient per degree, i.e., for each degree to be realized the number of occurrences and the average clustering coefficient per degree. For scaled replicas we scale the occurrences of all degrees by the scaling factor. This leads to the target number of nodes while also preserving the general shape of the degree distribution. In order to retain the distribution of the clustering coefficients, we leave them unchanged while scaling the network.

#### LFR

The LFR was designed as a benchmark graph generator for community detection algorithms (Lancichinetti et al. 2008). It generates graphs with a power law degree distribution and a community structure with community sizes that follow a power law distribution. Apart from the number of nodes, it requires parameters for power law distributions of the node degrees and the community sizes, and a mixing parameter that determines the ratio between intra- and inter-cluster edges.

We detect communities using Parallel Louvain Method (Staudt and Meyerhenke 2016) and fit the parameters for the two power law distributions as described above using the original degree sequence and the found community sizes. The mixing parameter is set to the ratio between intra- and inter-cluster edges of the found communities. As many networks in our set of real-world networks contain a few small connected components, the smallest communities usually only contain just two nodes while all other communities are much larger. Therefore, frequently our minimum power law exponent 1 is chosen and the expected average community size is still too low. In these cases we also fit the minimum community size using binary search until the expected average community size is the real average community size. Further, if the maximum community size is too small for the largest internal degree, we increase also the maximum community size to be 5% larger than the maximum internal degree. Even with these extra 5% we need several attempts for some of the graphs until random numbers are drawn that actually lead to fitting community sizes and node degrees. In Table 1 this is denoted by plfit*. For the scaling, we simply append copies of the same values.

## Computational experiments

Our implementations of ReCoN and the various fitting methods are based on NetworKit (Staudt et al. 2016), a publicly available tool suite for scalable network analysis. It also contains many of the generators we use for comparison and provides a large set of graph algorithms we use for our experiments. NetworKit combines C++ kernels with an interactive Python shell to achieve both high performance and interactivity, a concept we use for our implementations as well. All implementations are freely available as part of the package at https://networkit.iti.kit.edu. This also includes a faster and parallel implementation of the LFR generator (see below). Our experimental platform is a shared-memory server with 64 GB RAM and 2x8 Intel(R) Xeon(R) E5-2670 cores at 2.6 GHz; it runs on openSUSE Leap 42.2. We use the GCC 5.3.1 compiler for our C++ code. As described in Section Realistic replicas, we are interested in how well the different generators replicate certain structural features of the original networks as well as the running times of various graph algorithms. The results are described subsequently.

### Implementation details

**Fig. 3:**
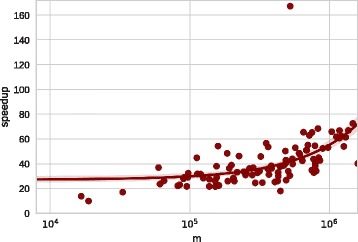
Speedup of NetworKit implementation of LFR compared to the reference implementation (Fortunato 2017) when replicating the set of 100 Facebook networks with *m* edges. Each point represents one network. The curve represents a linear regression model fit with its confidence interval (shaded area)

A reference implementation of the LFR generator by Fortunato et al. is available online (Fortunato 2017). We created a custom implementation of LFR in NetworKit, which is also the first parallelized implementation. Figure 3 illustrates the speedup that we achieve with a new implementation of the LFR generator based on NetworKit. It yields speedup factors of 18 to 70 (with an outlier of 167) for the test set of 100 Facebook social networks, and the factor grows superlinearly with the size of the network to be generated. The running time difference can be partially traced back to implementation differences: The LFR reference implementation relies on std::set to store and test for graph adjacencies, while our implementation uses a simple but apparently more efficient sequential scan on a simple array. Further, as mentioned already, the community graphs are generated in parallel, which gives an additional speedup.

For the tested generative models, NetworKit includes efficient implementations, such as implementations of linear-time algorithms for the Erdős–Rényi and Barabasi-Albert models (Batagelj and Brandes 2005) and a subquadratic time algorithm for Hyperbolic Unit Disk Graph model (von Looz et al. 2015). We have not implemented the BTER model; instead we use the FEASTPACK implementation by Kolda et al. (2014) with a “blowup” of 10 for a more accurate number of degree-1 nodes. NetworKit implements an adapter class that performs the model fitting and transparently calls the MATLAB-based FEASTPACK scripts using GNU Octave.

### Scaling behavior of the generators

The following experiments consider the scaling behavior of generative models. Given the parametrization discussed before, we look at the evolution of structural features with growing scale factor (*x*) up to *x*=32. We consider the same basic scalar features as for the real networks in “Realistic replicas” section.

**Fig. 4:**
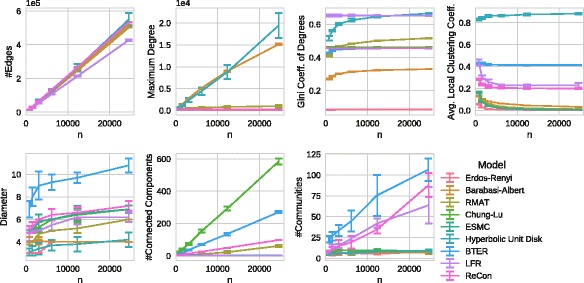
Scaling behavior of the different generators on the fb-Caltech36 network. From left to right and top to bottom: number of edges, max. degree, Gini coefficient of the degree distribution, average local clustering coefficient, diameter, number of components, number of communities. Each data point is the average over ten runs, the error bars show the standard deviation

In Fig. 4, we show the results of the scaling experiments for the fb-Caltech36 network. The number of edges of the replicas is increased almost linearly by all generators to ≈5·10^5^ edges, which approximately corresponds to 32 times the edges of the original network. Therefore, all generators seem to keep the average degree of the original network, which is expected as it is a parameter of all considered generators. Surprisingly, the maximum degree strongly increases up to 15 or 20 thousand with the Barabasi-Albert and the Hyperbolic Unit Disk Graph generators, respectively. The original maximum degree is 248, so that the new value is even significantly higher than the scaled maximum degree (i. e. 248 · 32). Actually, from the scaling study in “Realistic replicas” section, we could expect an increase, but rather in a lower range, so the degree distribution of the Barabasi-Albert and Hyperbolic Unit Disk Graph generators are not realistic in this regard. Concerning the Gini coefficient, one can clearly see that Erdős–Rényi model does not generate a skewed degree distribution at all (which does not come as a surprise, of course). All generators that get the exact degree sequence as input keep the Gini coefficient constant, which is expected and also relatively realistic from our scaling study. R-MAT shows a close fit of the Gini coefficient, too. LFR and Hyperbolic Unit Disk Graph have more skewed degree distributions while Barabasi-Albert has a less skewed degree distribution according to the Gini coefficient.

The original average local clustering coefficient of 0.43 is almost exactly reproduced by BTER, in which it is an input parameter. The Hyperbolic Unit Disk Graph method increases it to 0.8, most others obtain very small values. Only LFR and our new ReCoN generator are less far off with 0.25 and a slightly decreasing clustering coefficient; the latter is actually realistic as we saw in “Realistic replicas” section. Other generators produce much lower clustering coefficients.

The original diameter of 6 is closely replicated by many generators including ReCoN. BTER produces networks whose diameter is almost twice bigger. Barabasi-Albert and Hyperbolic Unit Disk Graph produce networks with lower diameters, this can be explained by their high-degree nodes that connect large parts of the network. All generators show a slight increase of the diameter when the networks are larger, which is consistent with our scaling study. But one should keep in mind that shrinking diameters have also been observed for some classes of complex networks (Leskovec et al. 2005).

While most generators produce networks with just a single connected component, Chung-Lu and BTER generate a large number, R-MAT and ReCoN a moderate number of connected components. In the case of Chung-Lu, BTER and R-MAT, this is probably due to a large number of degree-0 nodes. The original network consists of a giant component and three small components; ReCoN scales them linearly, which is due to its parametrization.

The original network is split into eight non-trivial communities, that number should increase slowly according to “Realistic replicas” section. Only in the networks generated by BTER, ReCoN and LFR, Parallel Louvain Method can find a significant and increasing amount of communities. While Parallel Louvain Method finds over 100 non-trivial communities in the network generated by BTER, there are fewer communities detectable in the networks generated by ReCoN and even less in the ones generated by LFR. Some deviation effects may certainly be due to the Parallel Louvain Method itself; but drastic differences do point to a strongly varying community structure.

Overall, ReCoN is the only generator that keeps the degree distribution, and produces a realistic clustering coefficient and a small diameter while keeping the graph connected and preserving a moderate number of communities. All other generators are either unable to keep the diameter or the connectivity or the number of communities. It is part of future work to investigate whether the full hyperbolic random graph model can alleviate the weaknesses of the unit-disk case.

In Appendix Appendix A: replicating structural properties we present results of further experiments on the preservation of different network properties both for scale-1 and for scale-4 replicas. The conclusion there is similar: for some properties, ReCoN is outperformed by other generators but overall it is able to produce the most similar replicas.

In Appendix Appendix B: DBLP scaling experiment we present a scaling study similar to the one of the Facebook networks but using the DBLP co-authorship network. Its increasing average degree over time is not captured by our current fitting methods. A further characteristic of the network, a high average local clustering coefficient combined with a low average degree is only captured by BTER, which is explicitly fitted with both properties. Both results show areas where ReCoN could be further improved.

### Realism in the running times of graph algorithms

Synthetic graphs are frequently used in algorithm engineering to estimate the running time of an algorithm assuming that this time will be similar on real networks. We examine if this is indeed the case with the generative models we consider. Using the previously described generators and fitting schemes, we generate replicas of 100 Facebook networks and test a variety of graph algorithms (see “Realistic replicas” section) on both the original and replica sets.

**Fig. 5:**
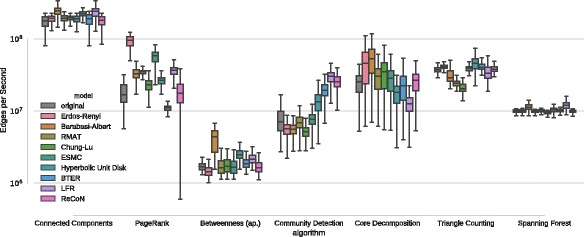
Running time replication of a set of network analysis algorithms. Running times are in edges per second, i.e., higher is faster

Our experiments demonstrate (see Fig. 5) that the running times on the replica sets often do not match those on the original set. The gray segments of the box plots represent the distribution of running times measured on the set of original networks. Ideally, the distribution on the synthetic networks would be identical. The difference is statistically nontrivial, though. Small variance between the models exists for connected components and spanning forest computations, since their running time is nearly constant per edge. Other algorithms exemplify how much running time can depend on network structure, especially community detection, core decomposition, triangle counting and PageRank. In general, the running time measurements obtained on ReCoN match the originals closely in most cases. An exception is community detection, where Parallel Louvain Method seems to profit from ReCoN’s explicit model of communities. BTER shows close matches, too.

### Generator running times

**Fig. 6:**
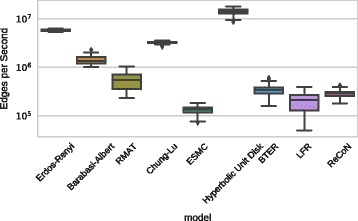
Fitting and generating: processing speed measured in edges per second (size of replica graph/total running time, measured on 100 Facebook graphs)

In Fig. 6, we show the running times of parameter fitting and generating a replica for all methods. Processing speed is given in the number of edges per second, a common measure for benchmarking graph algorithms that sets graph size and execution time in relation. The entire set of Facebook networks was used to produce the measurements, so generated replicas range from about 15000 to 1.5 million edges. For all models, generating the graph takes up the vast majority of time. The Edge-Switching Markov Chain model is the slowest on average, while the Erdős–Rényi and Hyperbolic Unit Disk Graph generators are the fastest. Our implementations of LFR and ReCoN are not among the fastest generators, but fast enough to produce millions of edges in minutes.

### Social network: example replication I

**Fig. 7:**
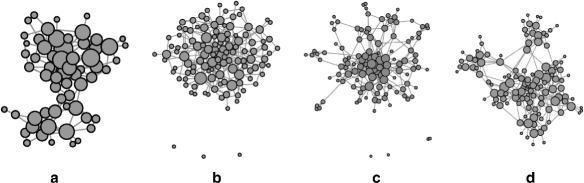
A small social network and its scale-2 replicas produced by different models. ReCoN is the model that best reproduces a set of essential properties, including degree distributions, clustering and community structure. **a** Original, **b** R-MAT with kronfit, **c** BTER, **d** ReCoN

As part of the realism evaluation, we replicate a small example network with each of the models. The input graph is a social network of Bottlenose dolphins. We use graph drawing with the ForceAtlas2 layout algorithm implemented in Gephi to inspect the network structure. Node sizes are proportional to degree within one drawing. Figure 7a shows a layout of the original network. Consider its basic structure: The graph is connected, with two distinct communities separated by an edge bottleneck. We also layouted replicas with scaling factors of 1 and 2 for each model, finding that the ReCoN replicas (e.g. Fig. 7d) are the only ones commonly recognized as resembling the original. BTER (Fig. 7c) is able to generate a similar community structure, though not matching the original as closely. All other replicas (e.g. those created by R-MAT after applying kronfit, Fig. 7b) lose the distinctive community structure of the original. While some effects may be due to the layout algorithm itself, we think that the results are clear enough to make the conclusions above.

### Epidemiological network: example replication II

**Fig. 8:**
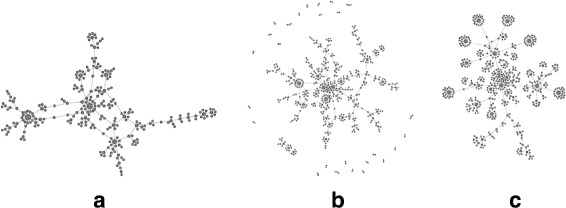
Colorado Springs epidemiological contact network. **a** original network, **b** scale-2 replica and **c** sample from a scale-200000 replica

An epidemiological network frequently used in studies on the transmission of HIV is based on data collected in Colorado Springs by Potterat et al. (2002). It contains 250 individuals who were in contact in the 1980s through sex or injection drug use. Figure 8a shows a force-directed layout of the network’s graph. Characteristic for this network is its tree-like structure and the presence of high-degree hubs with attached “satellite” nodes of degree 1. The Colorado Springs network is an instance of network data that cannot be shared freely due to legal restrictions, but no such restrictions apply to a synthetic replica. A replica made by the ReCoN generator reproduces structural features of the original with the highest accuracy among the considered models. More importantly, scaled replicas retain these essential properties, including the hub-and-satellite structure. Figure 8b shows the network replicated with a scaling factor of 2, which is remarkably close to the original. Like several other generators, ReCoN generates additional small connected components as an artifact. If this is undesirable, a postprocessing step could prune the network down to its giant connected component containing a large majority of nodes. (Interestingly, the original data set contained many singletons and isolated dyads that were removed from the graph in a preprocessing step).

Real epidemiological contact network data is difficult to collect, further complicated in the case of HIV by sex and drugs being tabu subjects. This makes obtaining such a network on the scale of an entire population impractical. In such a scenario, the ability to create realistic large synthetic replicas of smaller real networks may be highly relevant. As an explorative case study, we let ReCoN generate a replica of the Colorado Springs network with 50 million nodes, which corresponds to a scaling factor of 200, 000. Figure 8c shows a sample from a 5·10^7^ node replica. This network’s structure is quite different from the clustered “friendship networks” of dolphins and humans (Facebook). Remarkably, ReCoN also replicates many aspects of the original’s structure very closely, such as a tree-like structure with hubs and attached satellites. These aspects are retained even for huge scaling factors. This makes ReCoN a promising candidate to deliver large data sets in cases where large amounts of real data are likely unobtainable. Further domain-specific validation of the suitability of such replicas would be interesting.

## Multi-scale modeling and open problems

The ReCoN algorithm introduced above belongs to the class of single-scale generators in which the network structure is replicated in breadth in order to achieve an increased size synthetic network without considering structural properties at coarse-grained resolutions of the network. That is, the community structure is replicated multiple times, and connections are randomized while preserving key properties of the original network. The approach allows us to extrapolate from a given network to the next level up; however, it is clear that, as a network grows, its structure evolves new coarse-grained resolutions (or hierarchical levels) on top of those that were already present.

Progress in modeling such multiscale networks has been reported in (Gutfraind et al. 2015), which introduced a multiscale network generation approach, MUSKETEER. Briefly, starting from a single known or hypothesized network from any domain, MUSKETEER synthesizes ensembles of networks that preserve, on average, a diverse set of structural features *at multiple coarse-grained scales*, including degree, other measures of centrality, degree assortativity, path lengths, clustering, and modularity, while introducing unbiased variability across the ensemble in many of these properties. The core method is inspired by applications of the theory of multiscale methods (Brandt 2001; Briggs et al. 2000; Trottenberg et al. 2001) to combinatorial optimization (Brandt and Ron 2003; Meyerhenke et al. 2015; Ron et al. 2011). The original computational multiscale method starts with an optimization problem in connection with a network. It then (i) constructs a hierarchy of networks *G*_0_=*O*,*G*_1_,...,*G*_*k*_ that decrease in size via a coarsening procedure (Ron et al. 2011), (ii) solves a small optimization problem at the coarsest scale, and (iii) then iteratively uncoarsens the solution by gradual prolongations and local refinements from the coarse to next-finer scales. Similarly, in MUSKETEER a hierarchy of coarse networks is created; but, in contrast to the multiscale methods for computational optimization, nothing is optimized but the network is edited at all scales of coarseness. During the editing process only local changes are allowed, which are the results of local decisions only. In other words, the problem of network editing/replication/randomization is formulated and solved at all scales, where primitives at the coarse scale (such as coarse nodes and edges) represent aggregates of primitives at previous finer scale. Analogous to multiscale methods for computational optimization problems (Brandt 2001; Brandt and Ron 2003), by using appropriate coarsening, we are able to detect and use the “geometry” behind the original network at multiple scales, which can be interpreted as an additional property that is not captured by other network generation methods customized to replicate a small number of well known properties of *O* such as degree distribution. Moreover, it is known that the topology of many complex networks is hierarchical and thus might be produced through iterations of generative laws at multiple scales. In general, such generative laws often can be different at different scales, as evidenced by the finding that complex networks are self-dissimilar across scales (Carlson and Doyle 2002; Itzkovitz et al. 2005; Palla et al. 2005; Wolpert and Macready 2007; Binder 2008; Mones et al. 2012). These differences can naturally be reflected in the multiscale frameworks.

We believe that multiscale network modeling methods are particularly promising for addressing open problems we outline below. Some of the largest open problems are in the areas of statistical network hypothesis testing, network anonymization and compression, multiscale network decomposition and comparison, and understanding the fundamental limitations of network modeling.

### Statistical network hypothesis testing

One of the biggest unsolved problems in network science is the problem of hypothesis testing. This is a problem of high significance in fields such as infectious diseases as well as other research and its application. Given a network, the problem is to determine if the given network has statistically significant differences with a background network. For example, given a security problem on a network, could we say that a given network is significantly more “robust” (or any macroscopic property) than what would be expected by chance, where “chance” is defined realistically? The only existing approach, which is unsuitable for most applications, is to fit a parametric model such as an exponential random graph model (ERGM) to the network, and then test for the parameters being different from 0. This ERGM approach cannot be used in the vast majority of applications, because the network cannot usually be represented realistically using an ERGM, nor could most properties be represented easily using a generative parameter.

However, given the high realism and versatility achieved by multiscale network modeling methods, we believe that they, and non-parametric models more generally, could significantly advance statistical network hypothesis testing. The non-parametric method promises to provide a much more realistic permutational test, and therefore identify anomalous structures in a given network at a much lower level of false positives.

### Network anonymization and compression

A critical barrier towards advances in network science is the difficulty of sharing valuable real-world network data, which is often confidential and sensitive. This is the problem of network anonymization and compression. The multiscale strategy promises to address this gap through a powerful non-parametric data synthesis strategy, because, as we previously demonstrated, the strategy can generate networks that can model network structures with a high level of realism across a large spectral range. Initial success with such methods is possible already with the ReCoN and MUSKETEER strategies, but much work remains to be done addressing the spectrum of anonymization needs and developing versatile algorithms. Current algorithms operate with complete network exemplars or replicas, but it could be beneficial for anonymization to operate with only small-size descriptions of the network such as motifs or possibly global parameters.

The multiscale network modeling-based anonymization could be either non-parametric or parametric, allowing the user to limit the amount of data released to the public. In the parametric version, the data owner shares a controlled amount of network information, while the recipient is able to obtain a realistic representation of the data by using a multiscale algorithm.

Such a system would offer major advances in areas such as public health, cybersecurity and counter-terrorism. Data in these areas is often very difficult to collect and/or locked by privacy laws. Anonymization would compress the sensitive datasets and generate non-sensitive data, and in this form they could be shared with researchers in multiple institutions and even the public. This would vastly expand the number of people who can perform research in critical areas of scientific and practical interest.

### Multiscale decomposition and comparison of networks arising from real-world complex systems

The complexity of real network data suggests that its structure would be generally self-dissimilar when comparing several scales (or coarse-grained resolutions). However, existing network generators generally fail to address the problem of self-dissimilarity. For example, the degree distribution or the clustering coefficient of a communication network are not necessarily the same as those at its backbone at macro- and meso-scopic scales. Moreover, in the vast majority of practical cases, the coarse-grained structure is not given (or can be found) explicitly, which makes the attempts to generate realistic synthetic networks even more complicated. To address this problem, some of us and others have investigated a variety of multilevel optimization solvers for such problems, including graph partitioning (Buluç et al. 2016; Glantz et al. 2016; Meyerhenke et al. 2016), linear ordering (Ron et al. 2011), graph drawing (Meyerhenke et al. 2015), and node immunization (Leyffer and Safro 2013) in which the multiscale organization of a graph is expressed via low-energy cuts. However, other advanced approaches are needed when a decomposition into scales is not cut-based.

A related open problem has to do with the comparison of networks with self-dissimilar properties. In the existing multiscale algorithms like ReCoN and MUSKETEER, the comparison of the original and synthetic networks is done at the finest scale only. Whereas in some applications this can be acceptable, clearly, there are areas in which the multiscale structure is considered, and, thus, a multiscale comparison is much more beneficial and illuminating. However, to the best of our knowledge, there is no comprehensive concept of similarity that takes into account a comparison of properties at different scales.

### Fundamental limitation of network modeling algorithms

Despite many studies of network modeling, we appear to lack a fundamental theory to understand the limitations of these algorithms. There are at least three related problems. First, we lack a theoretical understanding for many of the most popular generating algorithms, including multiscale strategies such as MUSKETEER. Second, we lack a sense in which we can describe a network model or generator as in some sense “optimal” or “most realistic”. Perhaps it is possible to give an information-theoretic basis to understand the maximal realism a given generator can achieve given its parameter space. Third, complex networks are typically associated with a variety of different processes running on or creating them. Nearly all network generation models focus on rules that are structuring the network, overlooking the close dependence between the structure-forming processes and the processes on the network. Indeed, in many domains, the realism of a network is closely tied to the dynamics on the network. A domain expert (i.e., a potential user of network generation methods) cannot consider a synthetic graph realistic unless it becomes obvious that the processes that are supposed to co-exist with the network are also realistic (an issue we examined in the context of ReCoN). It is hoped that future studies in this field would examine this and earlier gaps in detail.

## Conclusion

We have presented a new generator, ReCoN, for replicating and scaling existing networks. In an extensive experimental evaluation we have shown that ReCoN is capable of generating networks which are (i) similar to the original network in terms of important structural measures and (ii) lead to similar running times of many graph and network analysis algorithms. Using ReCoN it is possible to realistically replicate an existing network, and to scale the synthetic version by orders of magnitude, e. g., in order to test algorithms on larger data sets where they are not available. Furthermore, it allows to create anonymized copies of such networks that can be distributed freely and allow to conduct representative experiments on them. While other generators sometimes perform better concerning certain criteria, none of the other generators is capable of approximately reproducing such a wide range of properties and running times.

We identified a better replication of local clustering coefficients as one of the areas where ReCoN could still be improved. Further, for certain types of networks such as co-authorship networks, fitting schemes for increasing average degrees could be investigated.

## Appendix A: replicating structural properties

In the existing literature on generative models, claims of realism are typically substantiated by showing that a set of structural properties is similar for real and synthetic networks. The large palette of properties to choose from and the question which of those properties are essential features makes this a complex problem. An often used approach is to describe a network by a feature vector, a set of scalar properties. These are often maxima, minima or averages of node properties (cf. the structural profiles published by the KONECT project (Kunegis 2013)). This can be reductionist, since these summary values may not give enough information about how the node properties are distributed. We therefore demonstrate how well the different models replicate networks with two types of plots. The first plot type shows scalar properties of the replicas, relative to those of the original. Let *x*_*o*_ and *x*_*r*_ denote scalar properties of the original and the replica, respectively. The relative value *x*_*r*_/*x*_*o*_ is computed for each replica. Box plots depict the distribution of these relative values over the entire set of replicas. Scalar properties included are: the number of edges, the number of connected components, the effective diameter (for 90% of node pairs) of the largest connected component, and the number of communities. The effective diameter is approximated using the ANF algorithm (Palmer et al. 2002). Communities are detected using the modularity-maximizing algorithm Parallel Louvain Method (Staudt and Meyerhenke 2016). This set of properties was chosen so that it could be quickly computed for a large set of networks. The second type of plot covers centrality measures, and is designed to show how the shape of the distributions of node centrality scores of the originals compares to those of the replicas. Each segment of the plot depicts the centrality values of all nodes of all networks in the considered data set or the replicas of a certain algorithm. Since centrality measures from this selection can have very different scales, all centrality scores are normalized to the interval [0,1].

**Fig. 9:**
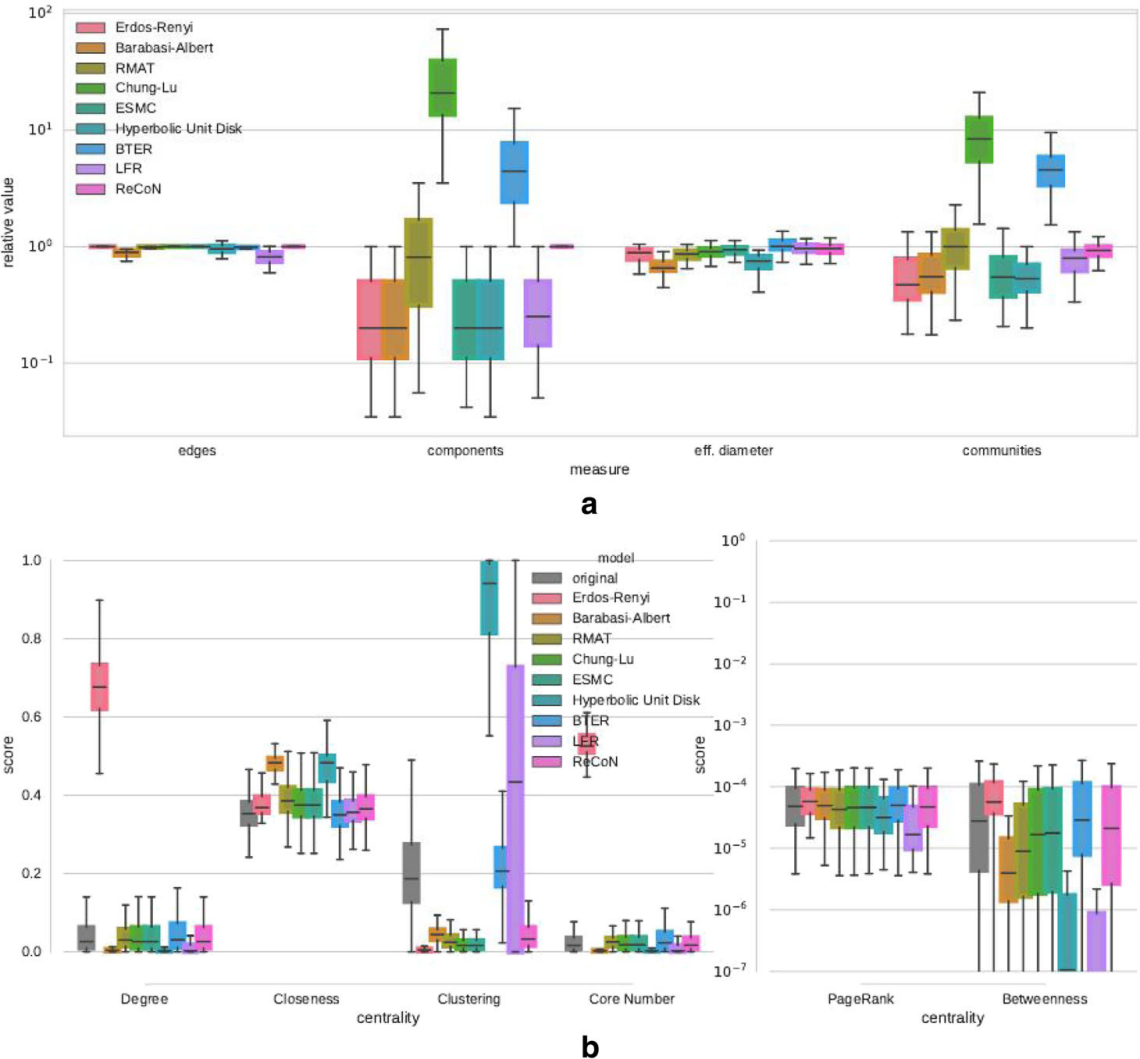
Structure replication of Facebook networks **a** Relative deviation of scalar network properties, **b** Distribution of centrality scores

Describing the results on 100 Facebook networks (Fig. 9a) from left to right, we observe the following results for the measured scalar properties: All models were parametrized to produce exactly *n* ^′^=*n* nodes, while some degree of freedom exists in the number of edges. However, all generators replicate the number of edges with a narrow variance, which is largest for LFR. ReCoN is the only model that matches the number of components with high accuracy, while it is lower for Erdős–Rényi, Barabasi-Albert, Edge-Switching Markov Chain, Hyperbolic Unit Disk Graph and LFR models, and significantly higher for Chung-Lu and BTER. The latter can probably be explained by the generation of isolated nodes instead of degree-1 nodes, which we have already seen in the visualizations before. There is no extreme deviation in terms of the effective diameter, but note that for small world networks, relatively small differences in the effective diameter may indicate significant structural differences. The Barabasi-Albert and Hyperbolic Unit Disk Graph models deviate the most and produce lower diameters. ReCoN, which receives a partition of the original into communities as input, replicates it closely, but all yield different numbers of communities. The Chung-Lu and BTER generators increase the number of communities by a factor of around 10, which is due to the larger number of connected components. Overall, ReCoN emerges with the most accurate replicas from this experiment.

The distributions of node centralities (Fig. 9b) compare as follows: All models except Erdős–Rényi are capable of producing skewed degree distributions, with Chung-Lu, Edge-Switching Markov Chain, BTER, R-MAT and ReCoN matching the original closely. Closeness is approximately matched by most models, but Barabasi-Albert and Hyperbolic Unit Disk Graph deviate significantly. The original networks feature a wide range of clustering coefficients, and only the BTER model, which receives explicitly this distribution, matches them exactly. For Hyperbolic Unit Disk Graph, clustering is extremely artificially high with a median close to 0.9, while LFR produces an unrealistically large variance. For the *k*-core numbers, random graphs are clearly outliers. R-MAT, Chung-Lu, Edge-Switching Markov Chain, BTER and ReCoN match well, while the very narrow distributions of the others point to a lack of differentiated *k*-core structure. Only small variations exist with respect to PageRank, and Hyperbolic Unit Disk Graph and LFR have strong deviations in terms of betweenness centrality, but interpretation is not straightforward in this case. In summary, extreme deviations in centrality score distributions clearly give away the artificiality of some synthetic graphs, such as the clustering coefficient for Hyperbolic Unit Disk Graph or the degree distribution for Erdős–Rényi. Other differences are more subtle, but possibly relevant. BTER replicates centralities most accurately.

**Fig. 10:**
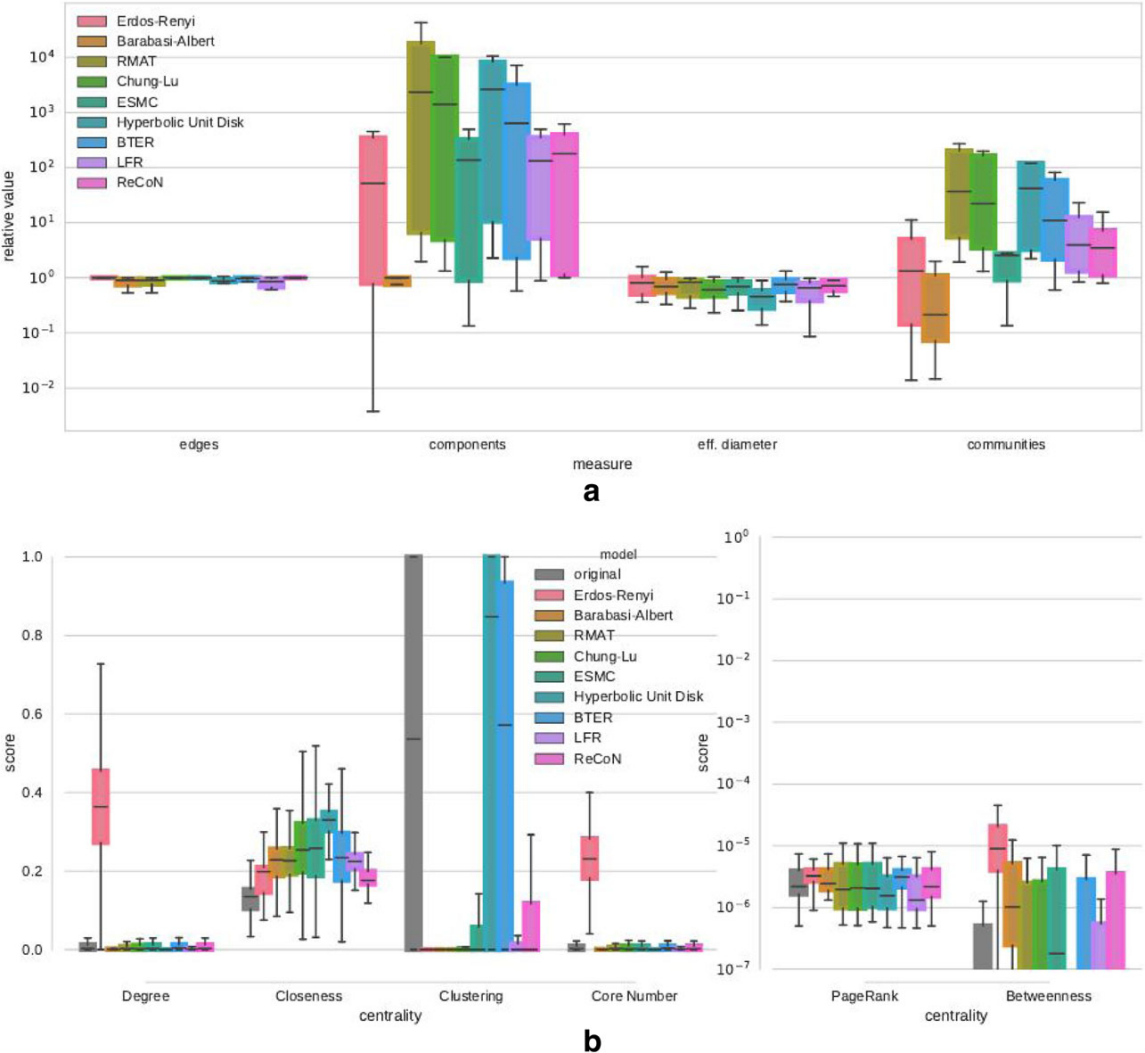
Structure replication of diverse set of networks (Table 2). **a** Relative deviation of scalar network properties, **b** Distribution of centrality scores

The results on a different set of networks (Table 2) are presented in Fig. 10. A notable difference is the extreme variance of clustering coefficients in the original set, which ReCoN cannot replicate. Again ReCoN performs well for the majority of properties.

**Table 2:** Additional networks used

Network	Type	*n*	*m*
Email-Enron	Email communication	36,692	183,831
PGPgiantcompo	PGP web of trust	10,680	24,316
As-22july06	Internet topology	22,963	48,436
Hep-th	Scientific coauthorship	8361	15,751
CoAuthorsDBLP	Scientific coauthorship	299,067	977,676
Dolphins	Animal social network	62	159
Power	Power grid	4941	6594
Cnr-2000	Web graph	325,557	2,738,969

The network email-Enron has been taken from the Stanford Large Network Dataset Collection (Leskovec and Krevl 2014), all other networks are from the clustering instances of the 10th DIMACS implementation challenge (Bader et al. 2014)

**Fig. 11:**
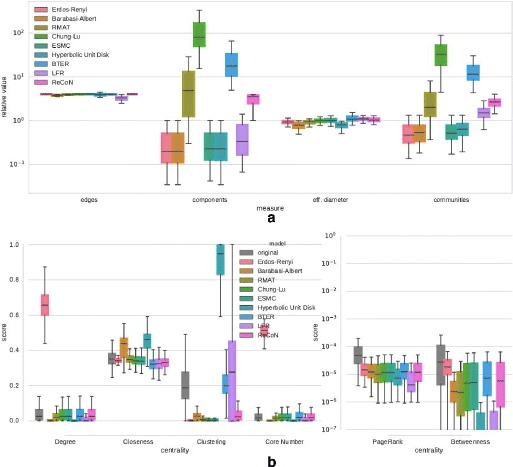
Structure replication of Facebook networks with scaling factor 4. **a** Relative deviation of scalar network properties, **b** Distribution of centrality scores

**Fig. 12:**
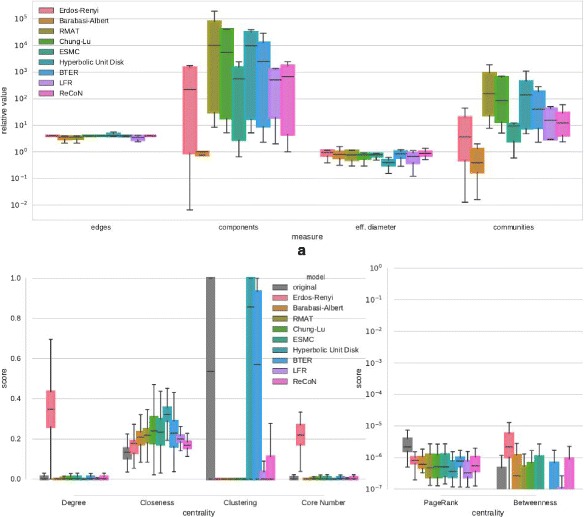
Structure replication of the diverse set of networks (Table 2) with scaling factor 4. **a** Relative deviation of scalar network properties. **b** Distribution of centrality scores

Figures 11 and 12 show results of a repetition of the experiment on the Facebook networks and the diverse set of networks with a scaling factor of 4. For the Facebook networks, the results for the scalar network properties in Fig. 11a can be summarized as follows: All models achieve the targeted edge factor of *m* ^′^=4·*m*. Chung-Lu and BTER have again an increased number of connected components. R-MAT and ReCoN show a slight increase in the number of connected components that corresponds approximately to the scaling factor of 4. For the effective diameter, small relative deviations matter: Barabasi-Albert and Hyperbolic Unit Disk Graph model tend to create smaller worlds than reality. ReCoN produces a remarkably exact match, considering that the generator does not explicitly target the diameter. It does, however, target a higher number of communities, which is desired and achieved. LFR keeps the number of communities constant on average, while many other models produce fewer communities than the originals. The relative deviations in the distributions of centralities are qualitatively equivalent to those in Fig. 9b. For the diverse set of networks, the properties of the scale-4 replicas in Fig. 12 are very similar to those of the scale-1 replicas in Fig. 10. The main differences are the scaled number of edges and also a larger number of communities for all networks.

**Fig. 13:**
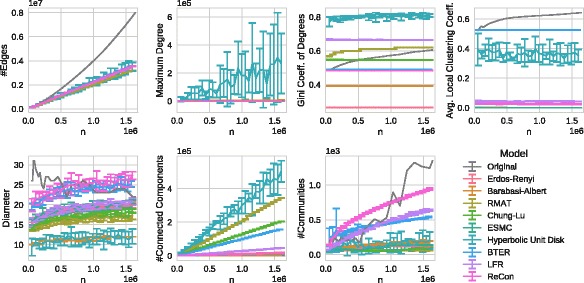
Scaling behavior of the different generators on the DBLP co-authorship network compared to the behavior of the original network over time. From left to right and top to bottom: number of edges, max. degree, Gini coefficient of the degree distribution, average local clustering coefficient, diameter, number of connected components, number of communities

## Appendix B: DBLP scaling experiment

In this section we examine the scaling behavior of the considered generators on a co-authorship network. For this, we use the DBLP co-authorship network as of July 3rd 2017, extracted using the tools provided by KONECT (Kunegis 2013). From this network, we consider annual snapshots starting with 1990 that contain data from all papers published until the end of that year. Of each of these snapshots, we only consider the largest connected component. We replicate the 1990 network using our set of generators up to a scaling factor of 29.

In Fig. 13, we demonstrate the properties of both the original network and the replicas. Concerning the number of edges, one can clearly see that the average degree of the DBLP graph increases over time while our fitting schemes assume a constant degree. The maximum degree is dominated by Hyperbolic Unit Disk Graph generator that produces very high maximum degrees. The Gini coefficient of the degree distribution of the original network is increased over time; this is also not modeled by most generators. R-MAT, however, actually produces graphs with increasing Gini coefficient. The average local clustering coefficient of the original network is very high compared to the low average degree, which is between 4.4 and 9.7. This is easily explained by the small clique that is formed by each paper. Only BTER, which explicitly replicates the local clustering coefficients, can replicate the high local clustering coefficient of the first snapshot. Even the Hyperbolic Unit Disk Graph generator does not reach such high clustering coefficients. The diameter of the DBLP graph is decreasing over time, which is not replicated by any of the generators. However, BTER and ReCoN produce graphs with similar diameter values. The graphs generated by Barabasi-Albert and Hyperbolic Unit Disk Graph have again lower diameter values. The number of connected components of the original is 1 as we selected only the largest connected components. As we have already seen for several other graphs, some of the generators produce a high number of connected components. Due to the low average degree, this is particularly dominant here. The number of communities found by Parallel Louvain Method on the original network is varying over time. ReCoN, but also LFR and BTER, generate community structures that have an increasing number of communities over time. However, none of them matches exactly the development of the original network, which also cannot really be expected. All in all, for this class of graphs BTER seems to be the generator that achieves the best replication results as it is the only generator that matches the average local clustering coefficient. Apart from the average local clustering coefficients, ReCoN is also able to reproduce all measured properties.

Many of the generators we consider could easily be adapted to support a non-linear edge scaling function. Also for ReCoN, scaling degrees is possible, though further work is needed to see how to deal with nodes whose scaled internal degree is larger than the number of available possible neighbors inside its community. Further, for this particular type of graph, fitting local clustering coefficients as it is done by BTER seems to be necessary in order to accurately replicate them. A possible extension of ReCoN could therefore be to take care of clustering coefficients while generating intra-community or inter-community graphs.
